# On the Use of Medicated Pessaries in the Treatment of Uterine Disease

**Published:** 1863-09

**Authors:** Thomas Hawkes Tanner

**Affiliations:** Assistant-Physician for the Diseases of Women and Children to King’s College Hospital, etc.


### ON THE USE OF MEDICATED PESSARIES IN THE TREATMENT OF UTERINE DISEASE.

By THOMAS HAWKES TANNER, M. D.,

*Assistant-Physician for the Diseases of Women and Children to King's College Hospital, etc.*

The great value of a variety of local applications in the treatment of uterine disease seems to be insufficiently appreciated by the profession at large. This is somewhat strange, considering the time which has elapsed since Dr. Simpson specially directed attention to their exceeding utility. Yet it is certain that a large number of practitioners know of no agents which may be directly applied to the uterus and vagina save the various kinds of caustics and injections. The latter are certainly of great benefit in many cases; but it must be allowed that, even when properly used, they are often of only temporary service, since they cannot be kept in contact with the diseased part for more than a few minutes.

The chief reason, I believe, for the non-employment of medicated pessaries has been the difficulty of so making them that they can be efficiently applied by the patient herself. When formed of certain drugs mixed into a mass with lard and wax they are either so soft that the sufferer cannot introduce them into the vagina; or, on the contrary, they are so hard that they fail to dissolve, and are expelled in just the same condition as that in which they were introduced. Although I have now employed these agents very freely for some years, I have found very few druggists who would take the trouble so to make them that they were of any service. The difficulties just alluded to have, however, been overcome since Mr. White Cooper directed attention ('Lancet,' 28th June, 1862) to the utility of the butter obtained from the *Theobroma Cacao* nut, from which chocolate is made, as the basis for ophthalmic ointments. This material possesses many valuable qualities, the chief being these:—It has an agreeable smell, and does not soil the fingers when handled; it does not become rancid; while, more particularly, though very firm, it has the property of becoming fluid at a low temperature. It is sometimes a little to stiff, but this fault is readily obviated by combining with it a little olive oil or glycerine. Pessaries made with cacao butter, though they have the consistence of wax while cold, are dissolved in the course of a few minutes when introduced into the vagina.

If ophthalmic surgeons are much indebted to Mr. White Cooper for directing attention to the uses of this butter, obstetric physicians are under no less an obligation. For although it had been used in America for making ointments for some time before this gentleman wrote of its merits, yet no physicians in this country were acquainted with its value, as far as I can learn from many inquiries.

The following formulæ far medicated pessaries are given as examples of the way in which I generally prescribe these remedies. It is only necessary to premise that rather large doses of the different drugs are necessary, inasmuch as absorption through the walls of the vagina is slow and uncertain:

1. *Iodide of Lead and Belladonna Pessaries.*—℞. Plumbi Iodidi, ℈ij; Extracti Belladonæ, ℈j; Butyri Cacao, ʒiv; Olei Olivæ, ʒj. Misce. Divide into four pessaries, and order one to be introduced into the vagina every night or every other night.

2. *Mercurial Pessaries.*—℞. Unguenti Hydrargyri, ℈iv—ʒij; Butyri Cacao, ʒiv; Olei Olivæ, ʒj. Misce. Where there is tenderness of the cervix uteri, one scruple of extract of belladona or two scruples of extract of conium should be added to the mass. Divide into four pessaries.

3. *Lead and Opium Pessaries.*—℞. Plumbi Acetatis, ℈j; Extracti Opii, gr. xij; Butyri Cacao, ʒiv; Olei Olivæ, ʒj. Misce. Divide into four pessaries. Order one to be used every other night.

4. *Zinc and Belladona Pessaries.*—℞. Zinci Oxydi, ʒj; Extracti Belladonæ, gr. xij—℈j; Butyri Cacao, ʒiv; Olei Olivæ, ʒj. Misce. Divide into four pessaries. One to be used every night.

5. *Iodide of Potassium and Conium Pessaries.*—℞. Potassii Iodidi, ʒj; Extracti Conii, ℈iv; Butyri Cacao, ʒiv; Glycerini puri, ʒj. Misce. Divide into four pessaries, and direct one to be used every night.

6. *Tannin and Catechu Pessaries.*—℞. Tanninæ, ℈ij; Pulveris Catechu, ʒj; Butyri Cacao, ʒiv; Olei Olivæ, ʒj. Misce. Divide into four pessaries, and order one to be used every other night.—*Trans. Obstetrical Society of London.*

